# A stunning new species of *Jamides* Hübner, 1819 (Lepidoptera, Lycaenidae), with notes on sympatric congeners from the Bismarck Archipelago, Papua New Guinea

**DOI:** 10.3897/zookeys.571.7356

**Published:** 2016-03-07

**Authors:** Chris J. Müller

**Affiliations:** 1 Honorary Associate, Australian Museum, 6 College Street, Sydney, NSW 2010; 2 Address for correspondence: PO Box 3228, Dural, NSW 2158

**Keywords:** Taxonomy, Lepidoptera, Lycaenidae, Polyommatinae, new species, Bismarck Archipelago, androconia

## Abstract

*Jamides vasilia* **sp. n.**, from montane West New Britain Province, Papua New Guinea, is described and illustrated. The new species is strongly divergent from other known *Jamides* Hübner, 1819 in possessing a high antenna-forewing length ratio, long androconia on the hindwing upperside and a strongly convex forewing inner margin in the male. It is compared by external structures, male genitalia and mtDNA sequence data to putative related species in the *cyta* group of *Jamides*. Notes on various *Jamides* taxa from the Bismarck Archipelago are also provided, with *Jamides pseudosias* (Rothschild, 1915) and *Jamides reverdini* (Fruhstorfer, 1915) recorded from New Britain for the first time.

## Introduction

*Jamides* Hübner, 1819, butterflies, commonly known as Caeruleans, belong to the subfamily Polyommatinae. The genus is distributed throughout much of the Oriental, Australian and Pacific region tropics (Rawlins et al. 2014) and comprises approximately 60 described species, with a concentration in South East Asia and New Guinea. (Hirowatari 1992) recognised 57 species and additional species have been described by Tennent and Rawlins (2012), and Takanami (1994). From approximately west to east, Kunte et al. (2015), Corbet and Pendlebury (1993), Treadaway and Schröder (2012), Rawlins et al. (2014), Parsons (1998), Tennent (2002) and Tennent (2006) identified six species for India, 16 for the Malay Peninsula, 16 for the Philippines, 21 for Maluku, 15 for Papua New Guinea, eight for the Solomon Islands and nine for the remainder of the South West Pacific, respectively.

Hirowatari (1992) proposed two groups and eight subgroups for *Jamides*. He noted that, together with members of the genus *Nacaduba* Moore, 1881, there is exceptional diversity of the male genitalia valva and their morphology is useful in placing species and making phylogenetic inferences about the group.

The new species introduced herein is exceptionally distinct from all other *Jamides* species. A number of other phenotypically distinct butterflies have recently been recorded from West New Britain Province (Müller 2013, 2014; Müller and Wills 2013), the type locality of the new species.

## Materials and methods

Examination of type and other relevant material was carried out in various institutions (as listed below). Adult specimens were photographed using a Nikon D300s Digital SLR Camera with a Nikon AF-S VR Micro-Nikkor 105mm f/2.8G IFED Macro lens and Nikon R1C1 Close-up Kit Flashes Speedlights. RAW images were edited using Adobe Photoshop CS6. Editing included alignment, auto contrasting and removal of background. A standardised procedure was followed with photography and image editing to ensure consistency of image output. Genitalia were extracted following maceration of abdomens in 10% KOH at room temperature for 36 hours. Genitalia were photographed in glycerol using the fore-mentioned camera body adapted to a Meiji Techno EMZ-5TR-P-FOI Trinocular Stereozoom Microscope, with OPTEK FL95E Fibreoptic Illuminator and twin arm optical fibre. Individual images were taken with the remote acquisition software DIYPhotoBits Camera Control 5.2. Sliced genitalia photographs were stacked and concatenated using the software Helicon Focus 6.0 and edited in Adobe Photoshop CS6. The plates were assembled in Adobe InDesign CS6 and the phylogeny tree in CorelDRAW X6. Genitalia were stored in small glycerol-filled vials pinned beneath the specimen.

Descriptions of external facies follow that of the numerical vein system of Corbet and Pendlebury (1993). Nomenclature of genitalic descriptions follows the same reference.

Tissue material (two legs) was collected from representatives of all of the eight *Jamides* subgroups of Hirowatari (1992), except that of the monotypic *celebica* Eliot, 1969 group, originally ascribed to *Epimastidia* Druce, 1891. Genomic DNA was extracted using the Qiagen DNEasy extraction kit, following the guided protocol by the manufacturer. Nucleotide sequence alignment was done by eye using Bioedit. For COI, a 654 bp fragment was amplified using Folmer et al. (1994) LCO (5'-GGTCAACAAATCATAAAGATATTGG-3') and HCO (5'-TAAACTTCAGGGTGACCAAAAAATCA-3').

Individual sequence properties were assessed using MEGA, version 4.1 (Tamura et al. 2006). Bayesian analyses of the dataset were carried out using MrBayes, version 3.0b4 (Ronquist and Huelsenbeck 2003). Three independent Bayesian runs at temperature settings in the range 0.2–0.4 were performed on the data using metropolis coupled Markov chain Monte Carlo simulations, from one to 5 million generations each, and tree sampling every 100 generations. Bayesian topology and branch posterior probabilities were computed by majority rule consensus after deleting the first 1000 000 generations (10 000 trees) as ‘burn-in’, after confirming that likelihood values had stabilized prior to the 100 000th generation.

Sequences were uploaded to GenBank (http://www.ncbi.nlm.nih.gov/genbank/) and are listed for each individual in the phylogenetic tree presented in Fig. 59. Additional sequences of *Jamides* taxa (also listed in the tree) were downloaded from Genbank and manually aligned with the dataset. Where possible, the species identity of the latter sequences was checked through the BOLD online database image gallery (http://www.boldsystems.org/).

### Abbreviations

AM
Australian Museum, Sydney, Australia 


CJMC
Reference collection of Chris J. Müller, Sydney, Australia 


MNHU
Museum für Naturkunde der Humboldt-Universität, Berlin, Germany 


NARI
National Agricultural Research Institute, Boroko, Port Moresby, Papua New Guinea 


NHM
Natural History Museum, London, England 


SMT
 Staatliches Museum für Tierkunde, Dresden, Germany 


## Taxonomy

### Jamides vasilia

Taxon classificationAnimaliaLepidopteraLycaenidae

Müller sp. n.

http://zoobank.org/2FF4EC45-5602-4583-BF61-424613106C50

Figs 1–9 , 46 , 53

#### Type material.

**Figures 1–15.**
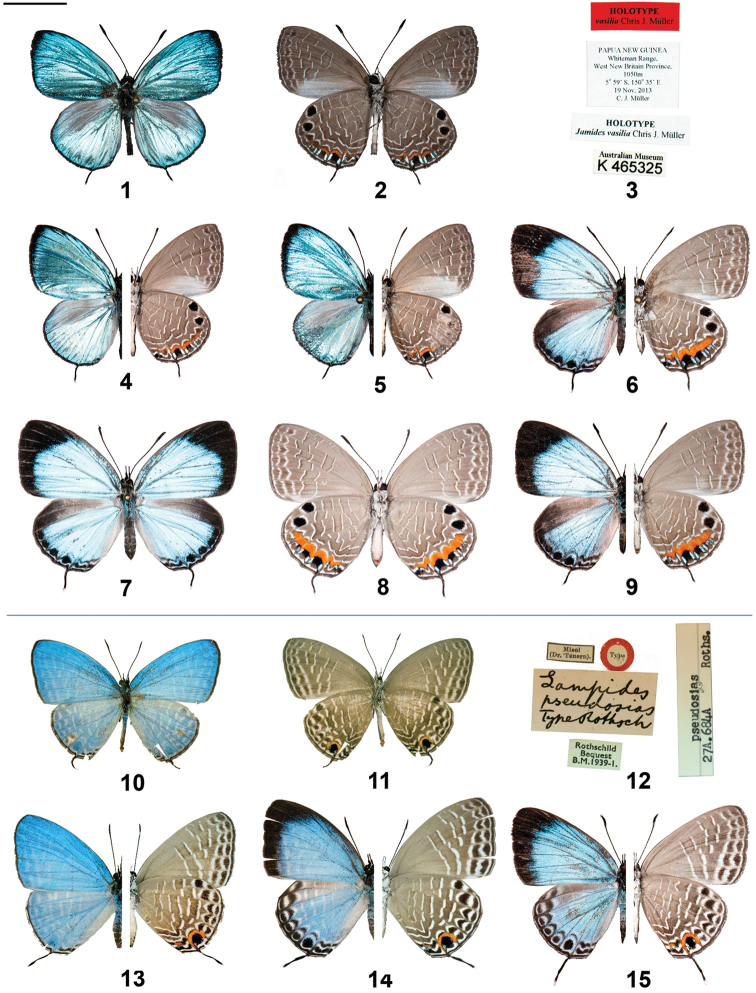
*Jamides* adults (left side upperside and right side underside, where halved) and label data. **1** *Jamides vasilia* holotype ♂ upperside **2** *Jamides vasilia* holotype ♂ underside **3** *Jamides vasilia* holotype ♂ label data **4** *Jamides vasilia* paratype ♂ (halved) **5** *Jamides vasilia* paratype ♂ (halved) (note rubbed hindwing) **6** *Jamides vasilia* paratype ♀ (halved) (note rubbed forewing) **7** *Jamides vasilia* paratype ♀ upperside **8** *Jamides vasilia* paratype ♀ underside **9** *Jamides vasilia* paratype ♀ (halved) **10** *Jamides pseudosias* holotype ♂ upperside **11** *Jamides pseudosias* holotype ♂ underside **12** *Jamides pseudosias* holotype ♂ label data **13** *Jamides pseudosias* ♂ (halved) (New Ireland) **14** *Jamides pseudosias* ♀ (halved) (New Ireland) **15** *Jamides pseudosias* ♀ (halved) (New Britain). Scale bar = 10 mm.

Holotype ♂ (Figs 1–3): Papua New Guinea, Whiteman Range, West New Britain Province, 1050m, 5°59'S, 150°35'E, 19 Nov, 2013, Chris J. Müller, genitalia dissected and held in vial pinned to specimen (AM), Registration: AM K.465325. Paratypes (3 ♂♂, 5 ♀♀): 1 ♂ labelled as holotype but dated 10 Nov, 2014 (NHM); 1 ♂ labelled as holotype but dated 26 Nov, 2014 (CJMC); 1 ♂ labelled as holotype but dated 22 Nov, 2014, Chris J. Müller (CJMC); 1 ♀ labelled as holotype but dated 10 Nov, 2014 (AM), Registration: AM K.465326.; 1 ♀ labelled as holotype but dated 13 Nov, 2014 (NHM); 1 ♀ labelled as holotype but dated 07 Dec, 2014 (CJMC); 1 ♀ labelled as holotype but dated 19 Apr, 2013 (CJMC); 1 ♀ labelled as holotype but dated 07 Nov, 2014 (NARI).

#### Diagnosis.

Both sexes of *Jamides vasilia* are highly distinctive and cannot be confused with any other known species. The uppersides of both sexes are brighter and more reflective than those of other species in the genus, the ground colour of the male in particular rivalling *Morpho* butterflies in intensity and radiance. The male of *Jamides vasilia* is unusual from a structural perspective, having long antenna (versus forewing length) that extend well beyond the cell and reach the postmedian area. The antenna/forewing length ratio is ~0.6, whereas in all other known *Jamides* species the antenna of the male is approximately half the length of the forewing. The shape of the male forewing is unique in that the tornus and inner margin are rounded, the latter being convex while in all other *Jamides* species the inner margin is straight. Also peculiar in the male is the large purple-brown patch occupying the costal one-third of the hindwing upperside. This patch is adorned with long androconial hairs (up to 5 mm in length). No other known *Jamides* bears such prominent structures. The male forewing upperside bears a thick terminal black border, tapering towards the tornus, not present in other species. On the underside, both sexes are easily recognised by the curved striae that are well displaced between veins on both wings and the two apical black spots on the hindwing are more rounded and pronounced than in other taxa. The area of orange bordered by metallic blue on the hindwing underside is extensive in both sexes, particularly the female, and extends from the inner margin all the way to space 5. The male forewing underside has the entire median area grey-white between the inner margin and vein 2.

The male genitalia of *Jamides vasilia* is unusual, bearing long teeth-like processes on the inner margin of the valva, with a spine-like process at the apex of the valva.

#### Description.

♂ Forewing length 15.8mm (Holotype), Antenna length 9.5mm (Holotype). Head grey; antenna long and extending well beyond end of cell, black ringed with white; thorax blue scaled on upperside, grey beneath; abdomen dark grey with blue scales near base on upperside; legs black with white between segments.

Forewing with tornus rounded (in congeners squared), and inner margin convex (in congeners straight).

Forewing upperside brilliant metallic sky blue, darkening slightly towards termen and apex, a prominent black termen border widening to ~1.5mm at apex; cilia black. Forewing underside deep grey-brown; inner margin broadly pale grey-white (in spaces 1a and 1b), with some metallic sky blue scales along vein 1b; termen narrowly white; a narrow grey-brown subterminal band, narrowly edged with white that forms triangular marks on basal edge; a postmedian band of similar colour to ground colour, about 2mm wide, narrowly edged with curved white on outside margin and with corresponding dark brown on inside margin, band is strongly displaced at veins by approximately 1mm, towards base in spaces 6 and 7 and incrementally towards base towards inner margin in spaces 2, 3 and 4; a median band at end of cell, approximately 1.5mm wide, curved and edged with white and dark brown as in postmedian band; cilia dark brown.

Hindwing rounded, with 2.5mm long, black tail at vein 2, tipped with white.

Hindwing upperside brilliant metallic sky blue, darkening slightly towards termen; a large purple-brown patch, clothed with long androconia along costa and in median area, occupying much of spaces 6 and 7 as well as part of cell and space 5; termen narrowly black; a series of diffuse black-dusted and indented subterminal spots, as well as two small black spots at tornus near intersection of vein 1b. Hindwing underside deep grey-brown; inner margin narrowly white, interspersed with black at vein ends; a large black apical spot (1.5mm diameter) in space 6 and another apical spot of similar colour and dimensions in space 7, both spots rimmed narrowly with white; two large subtornal black spots in spaces 2 (approx. 1mm across) and 3 (approx. 0.4mm across) and smaller, less regular black spots in spaces 1b and 4, each of these spots broadly edged along veins with metallic sky blue and basally with bright orange, and then fine white arcuate lines; an additional arcuate white subterminal line in space 5 linking the subapical and subtornal black spots; a postmedian band similar to that on the forewing underside, curved and strongly displaced at veins; a median band edged with white and dark brown at end of cell; a basal band approximately 1mm wide, edged narrowly with white, displaced at either side of cell; cilia dark brown.

♀: Forewing length 20.6mm, antenna length 10.8mm, antenna, thorax, abdomen and legs similar to male.

Forewing with inner margin straight.

Forewing upperside bright lustrous sky blue, darkening towards termen; apex of termen and apex broadly and sharply edged black (2.5mm and 8mm wide, respectively). Forewing underside similar to male, ground colour slightly paler and inner margin only narrowly white-grey and without blue scales, postmedian band extending to vein 1b, white edging to bands slightly wider and more diffuse.

Hindwing rounded, with black, white-tipped tail at vein 2 (approx. 4mm long).

Hindwing upperside bright lustrous sky blue, darkening towards termen; inner margin broadly black, basally transitional to brown, with narrow blue scaling along edge of space 6 and 7 adjacent to cell; a row of black subterminal spots (each averaging 0.6mm diameter) in spaces 2, 3, 4 and 5; two irregular black subtornal lines in spaces 1a and 1b, subparallel to termen; inner margin narrowly white. Hindwing underside similar to male, ground colour slightly paler, orange bordering subterminal spots wider and very extensive, reaching from the inner margin to space 5.

Male genitalia. Vinculum and tegumen ring broadly oval; sociuncus divergent; socii with lateral margin rounded, socii distinctly separated by straight, perpendicular sinus; saccus of even thickness, brachium widely bowed dorsally, yet sharply bent laterally, tapered dorsally; valva elaborate, hollowed, with serrated margin and teeth-like processes on inner margin, long narrow process stemming from near base of valva, weakly clubbed with pointed ventral surface on club; phallus with prezonal section approximately one tenth the length of postzonal section, slender, with apical rounded pencil-like process.

#### Etymology.

This enigmatic and exquisite new butterfly is named in honour of the author’s wife, Vasilia (Vicki) Savvas (Muller). Vicki has always supported the author’s obsession in butterfly research, despite the many sacrifices both on and off the field.

#### Distribution.

New Britain Island, Papua New Guinea.

#### Ecology.

Adults of *Jamides vasilia* inhabit moss forest and appear to have a more rapid, erratic flight than other members of the genus. Two females were initially observed flying around the base of a *Syzygium* R.Br. ex Gaertn. (Myrtaceae) sapling and resembled those of the lycaenid *Arhopala thamyras* (Linnaeus, 1758). The particular *Syzygium* plant had numerous, highly active, medium-sized brown ants present on the lichen-covered trunk but no early stages of *Jamides* could be located either on the foliage, trunk or in leaf litter surrounding the base of the plant. In the upper parts of the Whiteman Range (Figs 60, 61), *Jamides vasilia* flies with several other *Jamides* taxa, including *Jamides reverdini* (Fruhstorfer, 1915), *Jamides pseudosias* (Rothschild, 1915), *Jamides cyta* (Boisduval, 1832), *Jamides allectus* (Grose Smith, 1894), *Jamides soemias* Druce, 1891 and *Jamides amarauge* Druce, 1891. At lower elevations in the same mountain range, *Jamides celeno* (Cramer, 1775), *Jamides aetherialis* (Butler, 1884) and *Jamides nemophila* (Butler, 1876) are abundant.

#### Remarks.

The phylogeny of *Jamides*, presented in Fig. 59, comprises representative species from each of Hirowatari’s eight subgroups of *Jamides*, with the exception of the monotypic *Jamides celebica* (Eliot, 1969). In the Bayesian phylogeny, *Jamides vasilia* is recovered in a deeply diverged clade also comprising *Jamides cyta* and *Jamides nitens* Joicey & Talbot, 1916.

There is no significant variation in the type series of *Jamides vasilia*, with all specimens similar in size and shape. The male exhibits very slight variation in the width of the terminal black border.

The extent of distribution of *Jamides vasilia* on New Britain Island is not known. Based on the distribution of other endemic butterfly taxa on the island, it is unlikely to be restricted to the Whiteman Range, although no specimens have been observed during surveying, at a range of altitudes, of the Nakanai and Bainings Mountains, in central and east New Britain, respectively. *Jamides vasilia* appears to be a rare species.

### Notes on sympatric *Jamides* taxa in the Bismarck Archipelago

#### Jamides cyta

Taxon classificationAnimaliaLepidopteraLycaenidae

(Boisduval, 1832)

Figs 16–30 , 47 , 48 , 55

Catochrysops cyta : Boisduval (1832: 87); TL: New Ireland.

##### Remarks.

The type (?types) of *Jamides cyta* (Boisduval, 1832) were taken in New Ireland, during the voyage of the Astrolabe through the Indo-Pacific during the period 1826–1829. The Astrolabe, captained by Dumont d’Urville, visited at least three coastal sites in New Ireland; Port Praslin, Hèvre Cartret (Carteret Bay) and Likiliki in the Bay of Frondeurs (=Slinger’s Bay) (Domeny de Rienzi 1838). Port Praslin and Carteret Bay are separated by about 15km on the western side of New Ireland near the southern tip, in the Cape Saint George Channel. The location of Slinger’s Bay is given as “on the N. coast of New Ireland. Lon. 151. E. Lat. 3. S.” by Worcester (1817). The type/s of *Jamides cyta* have not been located. However, the description by Boisduval is clear, as follows:

“Ailes d'un bleu-argenté luisant; les inférieures avec une petite queue; dessous des quatre avec plusieurs raies blanches interrompues; les inférieures ayant en outré une rangée marginale de taches noires, don't les trois voisines du bord abdominal marquées de fauve et de vert doré.

Il a le port et la taille d’Elpis, auquel il resemble beaucoup.

Nouvelle-Irlande.”

Translated, this states that the insect (presumably a male), has “Wings a shiny silvery blue; the hindwings with a small tail; underside of the four [wings] with several broken white stripes; the hindwings additionally having a marginal band of black spots, of which the three adjacent [closest] to the abdominal [inner] margin are marked with fawn and golden green. It has the appearance and size of Elpis [*Jamides elpis* (Godart,[1824])], which it closely resembles. New Ireland.”

The description above is pertinent only to the male of *Jamides cyta*. The author has surveyed several sites throughout New Ireland, close to the localities visited by the Astrolabe, and found *Jamides cyta* to be particularly common at all of the lowland sites.

Toxopeus (1930), followed by Riley and Corbet (1938), recognised that *cyta* was the correct species name to be applied to the species known as *Catochrysops amphissa* (C. & R. Felder, 1860). Prior to that time, several subspecies of what are now known to represent *Jamides cyta* were described under *amphissa*, in particular several taxa from Maluku described by Fruhstorfer (1916). *Jamides cyta amphissa* is now known to be restricted to northern Maluku (Rawlins et al. 2014).

**Figures 16–30.**
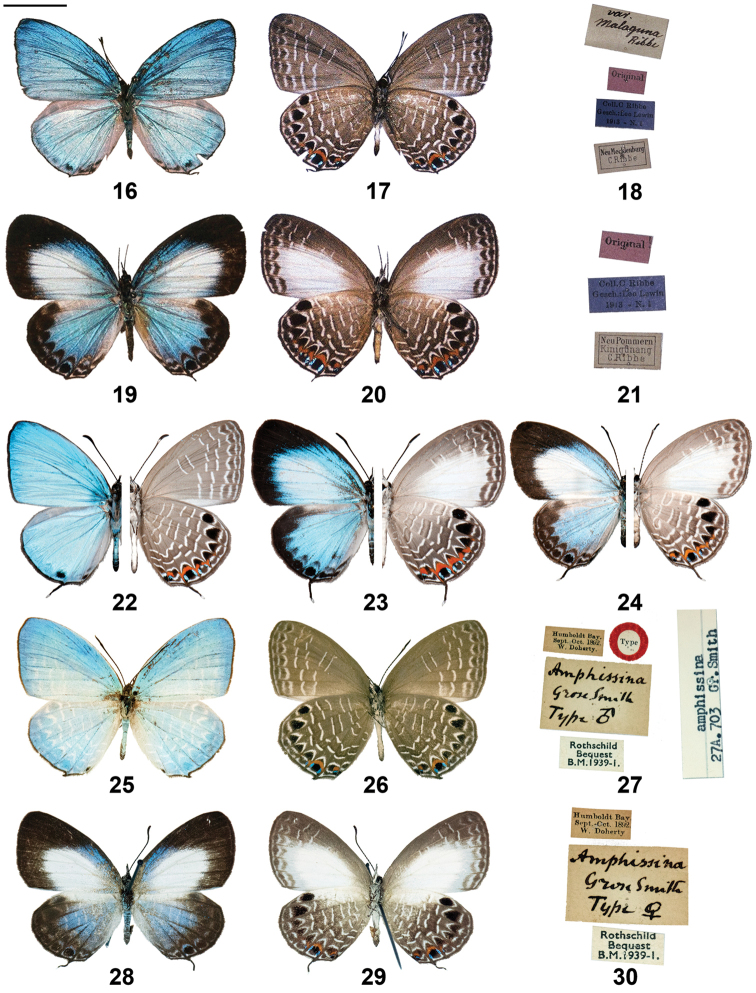
*Jamides cyta* adults (left side upperside and right side underside, where halved) and label data. **16** Lampides amphissina var. malaguna lectotype ♂ upperside **17** As Fig. 16, underside **18** As Fig. 16, label data **19** Lycaena amphissina var. malaguna paralectotype ♀ upperside **20** As Fig. 19, underside **21** As Fig. 19, label data **22** *Jamides cyta cyta* ♂ (halved) (New Britain) **23** *Jamides cyta cyta* ♀ (halved) (New Britain) **24** *Jamides cyta cyta* ♀ (halved) (New Ireland) **25** *Jamides cyta amphissina* holotype ♂ upperside **26** *Jamides cyta amphissina* holotype ♂ underside **27** *Jamides cyta amphissina* holotype ♂ label data **28** *Jamides cyta amphissina* allotype ♀ upperside **29** *Jamides cyta amphissina* allotype ♀ underside **30** *Jamides cyta amphissina* allotype ♀ label data. Scale bar = 10 mm.

Ribbe (1899: 228) proposed the name Lycaena amphissina var. malaguna for specimens collected in New Britain, Duke of York Islands and New Ireland. This taxon was considered by D’Abrera (1977: 355) to be a junior synonym of *Jamides cyta cyta* and Takanami (1989: 50) designated a male lectotype from New Ireland (Neu-Mecklenburg) and recorded three paralectotypes. Both the lectotype and a female paralectotype are here-in illustrated (Figs 16–21).

Nominate *cyta* from the Bismarck Archipelago is highly distinct from the New Guinea mainland subspecies *amphissina* Grose Smith, 1894 (not to be confused with *amphissa* from Maluku), in that the Bismarck specimens are larger and brighter, with much more pronounced orange on the hindwing underside. Bismarck *cyta* taxa may be further separated in that females from New Britain possess more extensive and brighter blue on the upperside than those from New Ireland and may warrant subspecific status. The types of *Jamides cyta amphissina* are illustrated in Figs 25–30 and both sexes of *Jamides cyta cyta* from the Bismarcks are also figured (Figs 22–24).

*Jamides cyta* is an easily recognised species, the male bearing a pale blue-white upperside and both sexes bear a row of triangular subterminal black spots on the hindwing underside. Note that the illustration of the male *Jamides* ‘*cyta*’ (as *Jamides* ‘*cytus*’) in Parsons (1998) is in fact that of *Jamides pseudosias*.

#### Jamides pseudosias

Taxon classificationAnimaliaLepidopteraLycaenidae

(Rothschild, 1915)

Figs 10–15 , 49 , 54

Lampides pseudosias : Rothschild (1915: 138); TL: Misol [Misool].

##### Remarks.

In the Bismarcks *Jamides pseudosias* was known only from one pair taken at an unspecified locality in New Ireland by A. F. Eichhorn in November 1923, which are held in the NHM (Figs 13, 14). A female taken at the type locality of *Jamides vasilia* is also figured (Fig. 15), representing the first record from New Britain.

*Jamides pseudosias* is readily distinguished from its congeners by its long hindwing tail, which is at least 1.5 times that of related species in New Guinea and by the rich blue colour of the male upperside. The forewings of the male are narrower and the apex more rounded than in related species. The forewing underside median bar and postmedian band are continuous across vein 3, though slightly oblique, giving the band a curved appearance. Note that Parsons (1998) illustrates a male of this species as that of *Jamides cyta* (*as J. ‘cytus*’; see under *Jamides cyta* in this work).

#### Jamides reverdini

Taxon classificationAnimaliaLepidopteraLycaenidae

(Fruhstorfer, 1915)

Figs 44 , 45 , 52 , 58

Lampides elpis reverdini : Fruhstorfer (1915: 143); TL: ST Holl. Zentral Neu Guinea, Kloofbivak [Papua, Indonesia].

##### Remarks.

*Jamides reverdini* was previously known from the Bismarcks from just a single specimen from New Ireland, which Parsons (1998) considered to be possibly mislabelled. Additional material is here recorded from close to sea level near Poronbus in New Ireland and from both the Whiteman Range (approximately 1000m) and Bainings Mountains (2000m), in West and East New Britain Provinces, respectively. Therefore, the taxon has a notably extensive vertical range in the Bismarck Archipelago. In northern mainland New Guinea, for example in the Upper Sepik, *Jamides reverdini* is most commonly observed below 500m (pers. obs.)

This insect is unique in its comparatively large size, bright silvery blue upperside and boldly patterned underside. On the underside, the ground colour is a distinct deep grey and, like *Jamides pseudosias*, the forewing median bar and postmedian band are connected at vein 3. *Jamides reverdini* is easily separated from *Jamides pseudosias* by, among other fore-mentioned features, the shape of the subterminal band on the hindwing underside. In *Jamides reverdini*, this band constitutes a series of rectangular markings bordered heavily with white, whereas in *Jamides pseudosias* these markings are distinctly triangular.

## Discussion

*Jamides vasilia* exhibits significant disparity from other members of *Jamides*. The male wing shape, with rounded tornus and convex inner margin, is unique, as is the black, tapering terminal border. On the forewing underside of the male the inner margin is broadly white-grey, relative to the remainder of the wing, a feature apparently unique in the genus. Also in the male, the hindwing upperside bears conspicuous androconia (Fig. 53), covering a purple-brown patch. Such prominent androconia are either absent or poorly developed in related species (Figs 54–58). The underside bands of both sexes are very curved and displaced at veins, unlike other *Jamides* taxa. *Jamides vasilia* is exceptionally bright, the male with the upperside luminescence possibly unmatched in Indo-Pacific Lycaenidae.

The male genitalia of *Jamides vasilia* (Fig. 46) are equally distinct and are unlike any of its congeners (Figs 47–52). The exaggerated teeth-like projections of the valva (Figs 46h, 46i) are unique. The dorsal projection of the valva is characteristic of the genus *Jamides* and in Indo-Pacific Polyommatinae appears to be shared only with the genus *Callictita* Bethune-Baker, 1908.

**Figures 31–45.**
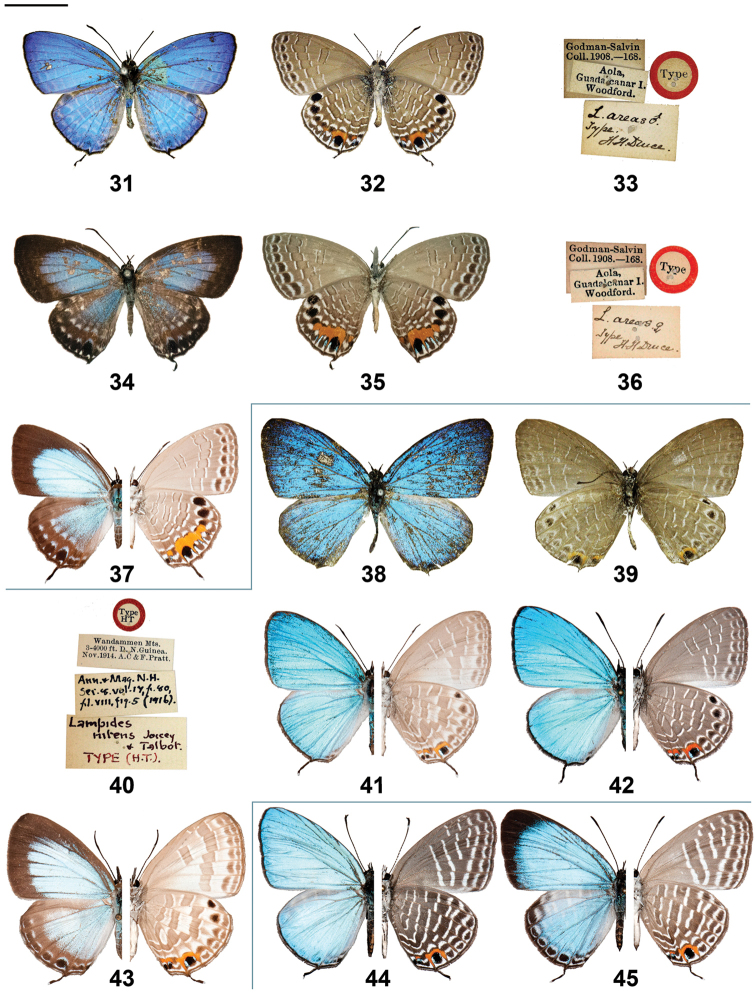
*Jamides* adults (left side upperside and right side underside, where halved) and label data. **31** *Jamides areas* holotype ♂ upperside **32** *Jamides areas* holotype ♂ underside **33** *Jamides areas* holotype ♂ label data **34** *Jamides areas* allotype ♀ upperside **35** *Jamides areas* allotype ♀ underside **36** *Jamides areas* allotype ♀ label data **37** *Jamides areas* ♀ (halved) (Guadalcanal) **38** *Jamides nitens* holotype ♂ upperside **39** *Jamides nitens* holotype ♂ underside **40** *Jamides nitens* holotype ♂ label data **41** *Jamides nitens* ♂ (halved) (Telefomin) **42** *Jamides nitens* ♂ (halved) (Mianmin Range) **43** *Jamides nitens* ♀ (halved) (Telefomin) **44** *Jamides reverdini* ♂ (halved) (New Britain) **45** *Jamides reverdini* ♀ (halved) (New Britain). Scale bar = 10 mm.

**Figures 46–48.**
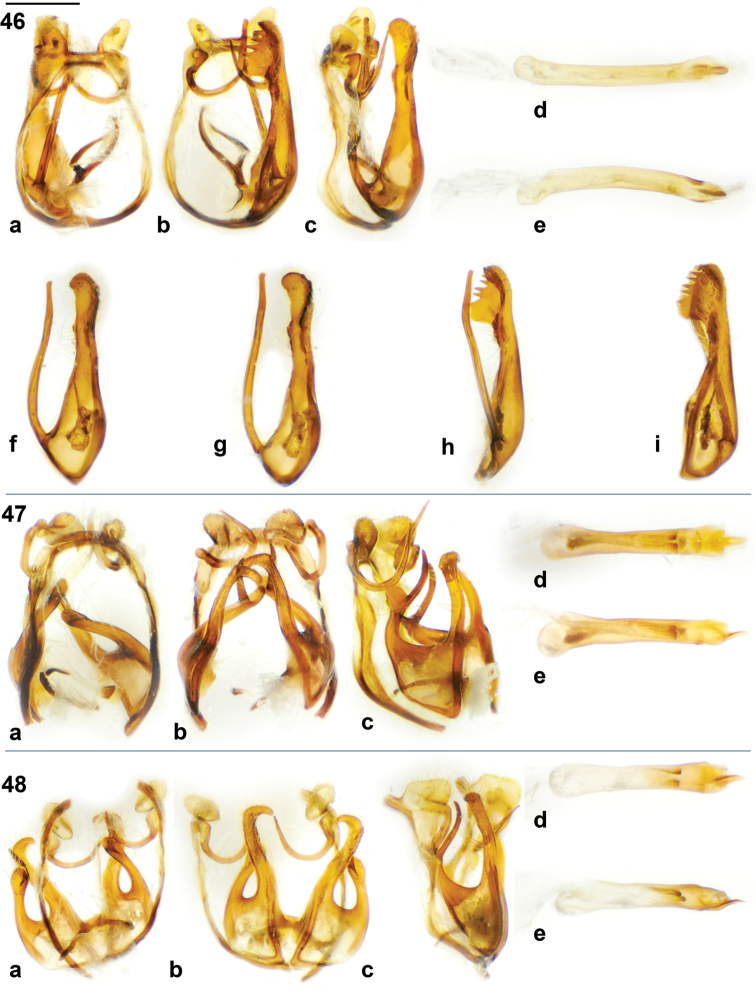
*Jamides* male genitalia. (**a** genitalia in dorsal view with aedeagus removed, **b** genitalia in ventral view with aedeagus removed, **c** genitalia in lateral view with aedeagus removed, **d** aedeagus in dorsal view, **e** aedeagus in lateral view, **f** valva in lateral external view, **g** valva in lateral interior view, **h** valva in dorsal view, **i** valva in ventral view. **46** *Jamides vasilia* holotype ♂ **47** *Jamides cyta cyta* (New Britain) **48** *Jamides cyta amphissina* (Sepik, mainland NG). Scale bar = approx. 1 mm.

**Figures 49–52.**
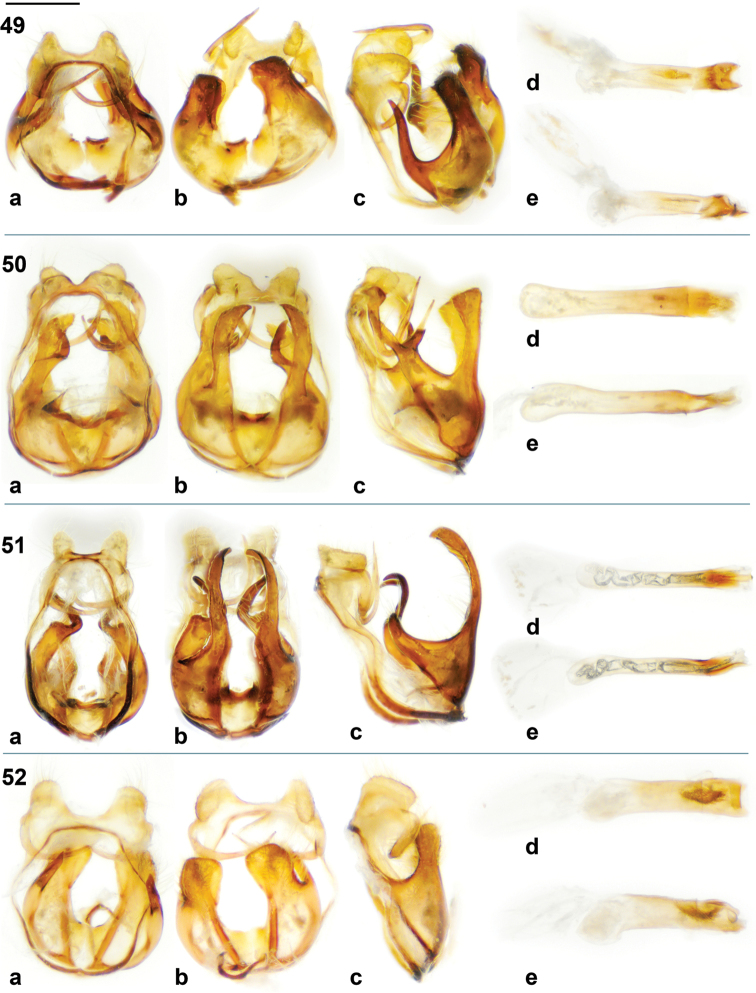
*Jamides* male genitalia. (**a** genitalia in dorsal view with aedeagus removed, **b** genitalia in ventral view with aedeagus removed, **c** genitalia in lateral view with aedeagus removed, **d** aedeagus in dorsal view, **e** aedeagus in lateral view. **49** *Jamides pseudosias* (Mianmin Range) **50** *Jamides areas* (New Georgia, Solomons) **51** *Jamides nitens* (Mianmin Range) **52** *Jamides reverdini* (New Britain). Scale bar = approx. 1 mm.

**Figures 53–58.**
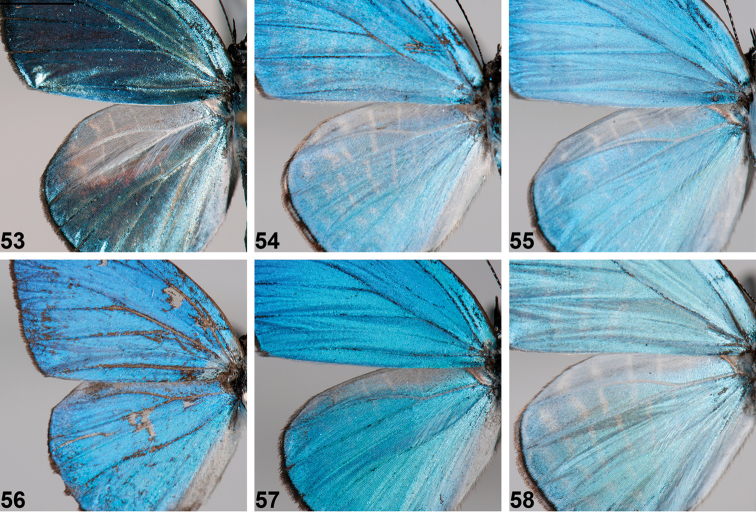
*Jamides* male hindwing apex, showing development (or absence) of androconia. **53** *Jamides vasilia* paratype ♂ **54** *Jamides pseudosias* (Mianmin Range) **55** *Jamides cyta cyta* (New Britain) **56** *Jamides areas* (New Georgia, Solomons) **57** *Jamides nitens* (Mianmin Range) **58** *Jamides reverdini* (New Britain). Scale bar = approx. 1 mm.

That *Jamides vasilia* was phylogenetically recovered, as a result of Bayesian Inference, within a clade of *Jamides* also comprising *Jamides cyta* and *Jamides nitens* is perhaps unexpected, considering that *Jamides vasilia* is phenotypically very different from either of these taxa. Indeed, there is significant genetic divergence (in COI barcode) between *Jamides vasilia* and *Jamides cyta* or *Jamides nitens*. However, a potential similarity between *Jamides vasilia* and *Jamides cyta* is that the latter has been recorded feeding on *Syzigium* in Australia (Braby 2000) and, based on adult female behaviour (see Remarks section), is possibility the food plant of *Jamides vasilia*. *Jamides nitens* (Figs 38–43) is one of the, otherwise, brightest *Jamides* taxa. Parsons (1998) noted that *Jamides nitens* exhibits a deeper blue than related species in Papua New Guinea. Although the male upperside rich sky blue appears to be constant in all specimens, both sexes exhibit some degree of variability of the amount of white suffusion bordering the striae on the underside (hence three males of *Jamides nitens* are figured, see Figs 38–42).

*Jamides vasilia* is a montane species, having been recorded from 850 to 1050m. An additional female was observed at 700m in the Whiteman Range. Of the related *Jamides* taxa, *Jamides nitens* is also a montane insect, Parsons (1998) recording it from 1200–1800m, although the author has recorded this species from the Mianmin Range from as low as 550m. *Jamides cyta* is typically a lowland species and is rarely recorded above about 500m, although it has been seen very occasionally up to 900m in the Whiteman Range (pers. obs.). Parsons (1998) records *Jamides cyta* from sea level to 700m. *Jamides reverdini* and *Jamides pseudosias* appear to be predominantly montane in New Britain, the former being seen by the author at over 2,000m in the Bainings Mts, East New Britain Province. However, in mainland New Guinea, both species tend to occur in the lowlands. In the Solomons, *Jamides areas* (Figs 31–37) is generally a lowland species and the species was found to be fairly common at Uepi Island, New Georgia Group, at sea level during 1986 (pers. obs.). It is unknown to what extent, if any, vertical elevation separation has played in the role of *Jamides* diversification in the New Guinea region.

Various phylogenetic analyses produced a range of trees for the *Jamides* dataset. Indeed, in several, including Neighbour-Joining, Maximum Likelihood and Maximum Parsimony, the resultant tree suggested that *Jamides vasilia* is sister to all assessed *Jamides* species groups and that it may represent a separate genus. Given the variable results from the different methods, COI alone is clearly not an adequate tool and additional sequencing of nuclear gene fragments would undoubtedly assist in better resolving the phylogenetic position of the new taxon.

**Figure 59.**
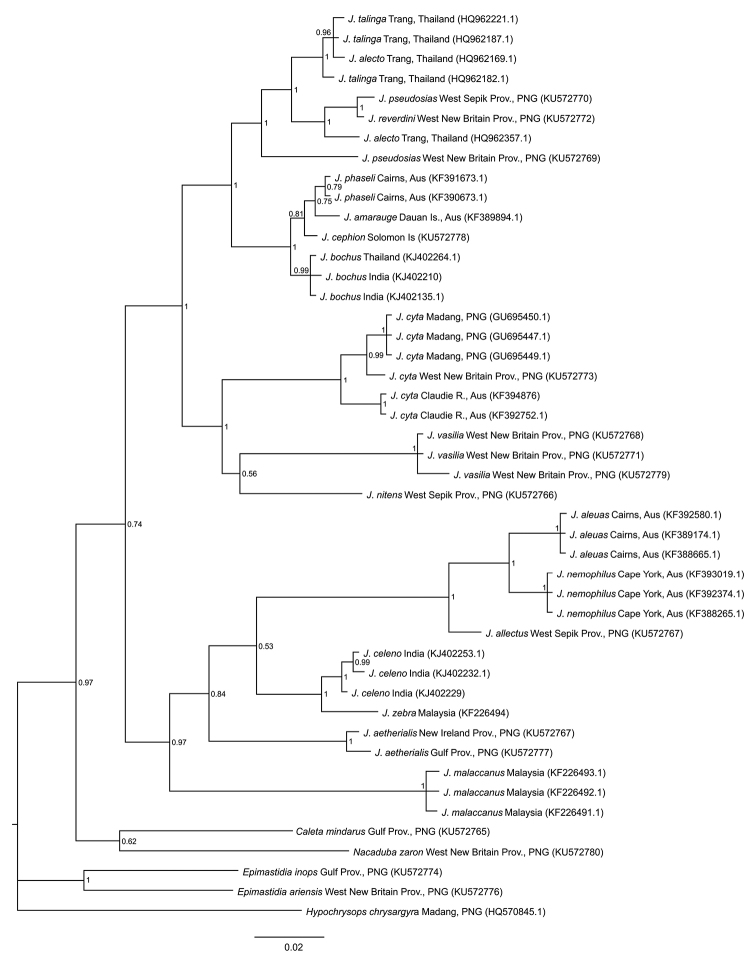
Fifty percent majority rule consensus phylogram for selected *Jamides*, from a Bayesian analysis of 650 base pairs of COI gene fragment. Numbers at the nodes are the posterior probabilities of those nodes. The scale bar represents 2% genetic distance. Each terminal indicates the taxon name, locality and Genbank sequence accession number. Note the following abbreviations in parentheses for Province (Prov.), Papua New Guinea (PNG) and Australia (Aus).

**Figure 60.**
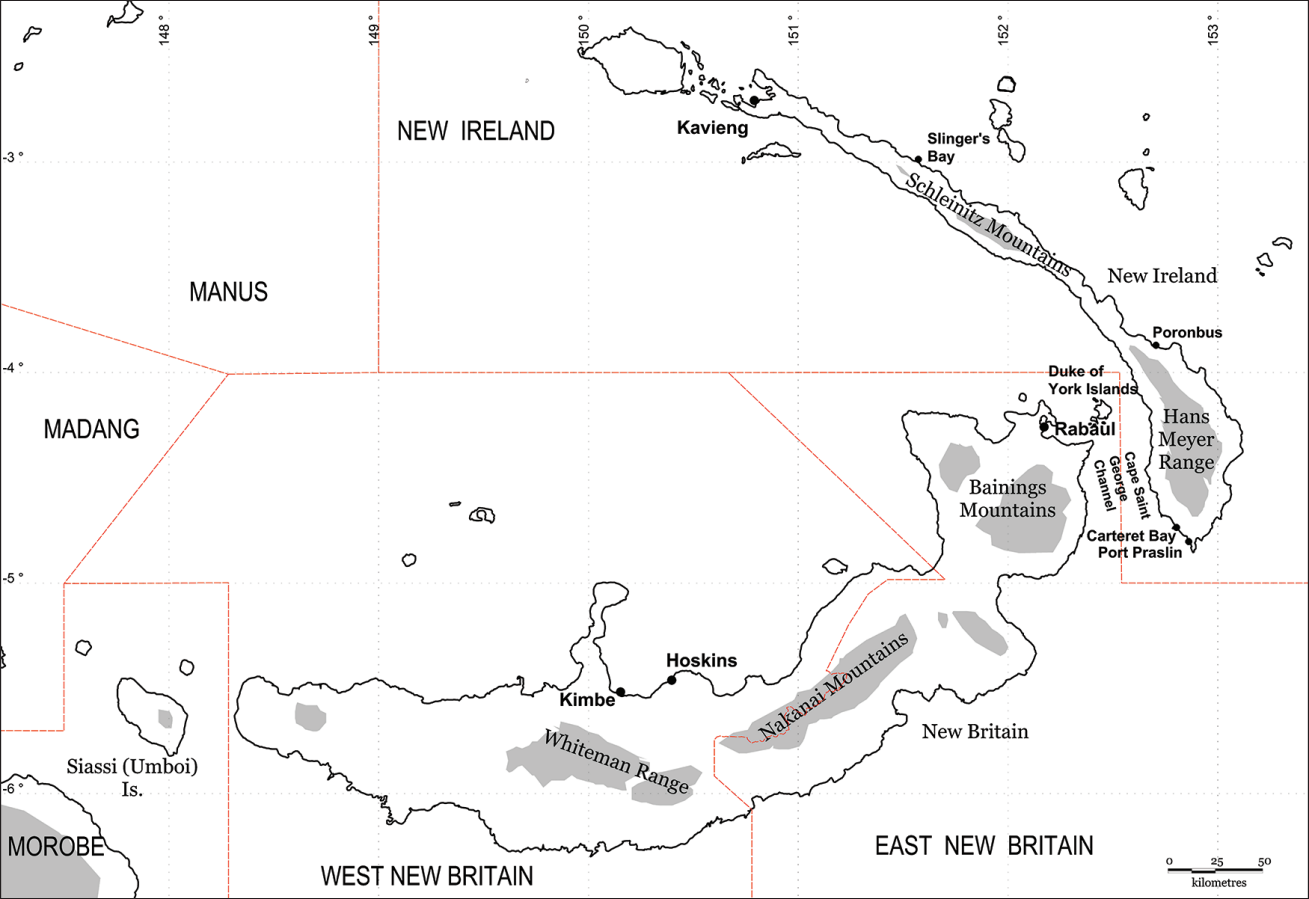
The Bismarck Archipelago, Papua New Guinea, showing all main islands (excluding the Admiralty Group), provinces (upper case), main centres and localities referred to in the text. Shading represents approximate expanse of land above 1000 m elevation.

**Figure 61.**
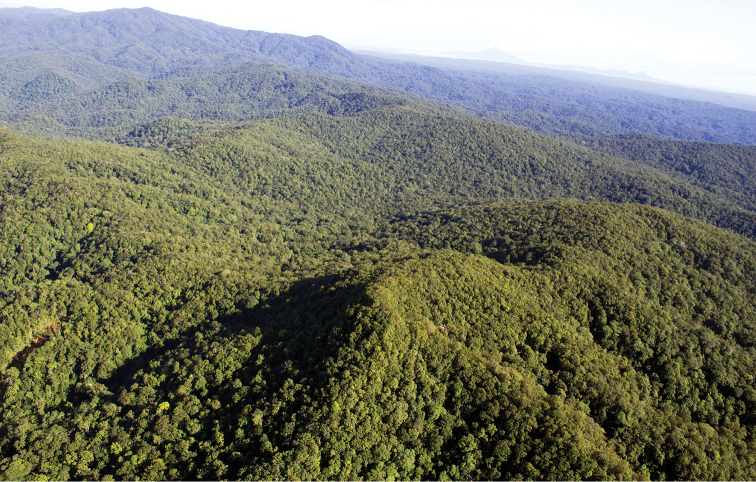
The Whiteman Range, West New Britain; type locality of *Jamides vasilia*.

## Supplementary Material

XML Treatment for Jamides vasilia

XML Treatment for Jamides cyta

XML Treatment for Jamides pseudosias

XML Treatment for Jamides reverdini
